# Moderate Alcohol Consumption Increases the Risk of Clinical Relapse in Male Depressed Patients Treated with Serotonin-Norepinephrine Reuptake Inhibitors

**DOI:** 10.3390/diagnostics14111140

**Published:** 2024-05-30

**Authors:** Mădălina Iuliana Mușat, Felicia Militaru, Victor Gheorman, Ion Udriștoiu, Smaranda Ioana Mitran, Bogdan Cătălin

**Affiliations:** 1 U.M.F. Doctoral School Craiova, University of Medicine and Pharmacy of Craiova, 200349 Craiova, Romania; madalina.musat3@gmail.com; 2 Experimental Research Centre for Normal and Pathological Aging, University of Medicine and Pharmacy of Craiova, 200349 Craiova, Romania; bogdan.catalin@umfcv.ro; 3 Department of Psychiatry, University of Medicine and Pharmacy, 200349 Craiova, Romania; feliciobanu@yahoo.com (F.M.); gheormanv@gmail.com (V.G.); 4 Department of Physiology, University of Medicine and Pharmacy of Craiova, 200349 Craiova, Romania

**Keywords:** depression, antidepressants, alcohol, liver enzymes, NAFLD

## Abstract

Background: While depression can be associated with multiple comorbidities, the association between depression and liver injury significantly increases the mortality risk. The aim of this study was to evaluate if moderate alcohol intake affects the rate of clinical relapses in patients treated with antidepressants as monotherapy. Methods: We assessed, over a period of 30 months, the clinical records of 254 patients with depressive disorder, of either gender, without additional pathologies, receiving monotherapy treatment with antidepressants. Thirty-three patients with alcohol abuse, alcoholism or significant cognitive impairment were excluded. The medical and psychiatric history, medication and liver enzyme values were collected and analyzed. Results: Out of the 221 patients who met the inclusion criteria, 78 experienced relapses of depression. The rate of relapse did not correlate with the levels of liver enzymes. Alcohol consumption, as objectified based on GGT levels and the AST/ALT ratio, suggested that men had higher alcohol intake compared to women. Patients treated with serotonin-norepinephrine reuptake inhibitors (SNRIs) with elevated AST levels were approximately 9 times more likely to relapse, while the ones with elevated GGT had a 5.34 times higher risk. While GGT levels remained a marker for relapse in men with elevated GGT, ALT and not AST proved to be a better risk indicator for relapses in male patients. Conclusion: The use of SNRIs in depressed male patients with moderate alcohol intake should be carefully considered, as they might be susceptible to higher risks of relapse compared to alternative antidepressant therapies.

## 1. Introduction

Depression is a leading cause of disability worldwide in the adult population [1] and is associated with significant morbidity and mortality [2]. Despite the availability of pharmacological treatments, nearly 60% of patients do not achieve recovery after undergoing a single trial of medication [3]. Untreated or poorly managed depression can have serious consequences, impairing a person’s ability to function in daily activities [4], work [5], relationships [6] and overall quality of life [7]. Worldwide, it is estimated that every 40 s, one person commits suicide [8]. About 60% of suicides are committed by people with major depressive disorder (MDD) [9], making it one of the most common causes of death [10].

MDD is also associated with various health problems, such as hypertension [11], diabetes [12], coronary artery disease [13] and nonalcoholic fatty liver disease (NAFLD) [14]. When depression and NAFLD are associated, there is an increased likelihood of complications and mortality [15]. With depression increasing, by up to 50%, the risk of NAFLD [16] and the antidepressant (AD) treatment often inducing liver injury (DILI) [17,18,19,20], the need for monitoring the liver function in depressed patients becomes apparent [21,22]. While an elevated value of alanine aminotransferase (ALT) serves as a reliable indicator of hepatocellular damage resulting from NAFLD, especially in males [23], and the hepatic toxicity induced by ADs [24], gamma-glutamyl transferase (GGT) levels typically rise in response to excessive alcohol consumption, making it a valuable biomarker for assessing alcohol-related liver damage and monitoring alcohol intake in clinical settings [25,26,27].

When alcohol consumption is associated with depression, managing the clinical outcome becomes difficult, as the relation between alcohol and depression is interdependent [28], with most of the patients experiencing both conditions [29]. Alcoholic patients with associated depression face the highest risk of relapse and returning to alcohol consumption to relieve depression symptoms [30]. With women being more susceptible to develop symptoms of depression [31], alcohol intake patterns also differ between genders. Men still generally consume more alcohol than women, but nowadays, the gaps are narrowing [32]. Previous studies report higher rates of alcohol use in men, including binge drinking [33,34] and alcohol dependence [35]. Biological factors, such as differences in body composition [36] and metabolism [37], may partially explain these discrepancies. Additionally, cultural norms and socialization processes may influence alcohol consumption behaviors differently for men and women [38]. Therefore, an appropriate therapeutic approach is essential, as the presence of alcohol consumption is associated with higher treatment refractivity [39]. Choosing the right antidepressant requires a careful evaluation, encompassing all individual aspects of the patients, as well as their habits. The majority of prior research has primarily focused on the ADs prescribed for individuals with a history of alcohol abuse [40,41,42,43], and although some ADs have proven effective [44,45], the overall outcome seems to depend on the degree and severity of alcohol dependence, with better results being reported for lower-risk patients [46,47]. Therefore, the anticipated outcome is contingent upon substantial or moderate alcohol intake, so consideration must be given to the potential complications that can occur even with what is considered moderate alcohol consumption.

In the present study, we aimed to assess the impact of moderate alcohol consumption, as observed through a liver biological evaluation, on the likelihood of clinical relapse in patients treated with different antidepressant treatments.

## 2. Materials and Methods

### 2.1. Sample

The clinical records of 254 patients were retrospectively–prospectively assessed over a period of 30 months (1 January 2021–31 June 2023) in the Psychiatry Clinic I-Neuropsychiatry Hospital of Craiova. At the time of hospitalization, informed consent was obtained from all participants. The retrospective aspect involved the thorough examination of past clinical records to gather relevant data. In the prospective component of our study, we specifically focused on patients who experienced relapsing episodes during the study period. We defined relapse episode as the occurrence of another depressive episode within six months following the acute phase of depression [48]. The study was conducted according to the guidelines of the local Ethics Committee of the University of Medicine and Pharmacy of Craiova (no. 67/20.04.2022) and the Ethical Council of Neuropsychiatry Hospital of Craiova (no. 2/02.05.2022), under Romanian and European laws, in accordance with Helsinki ethical guidelines.

### 2.2. Inclusion and Exclusion Criteria

**Figure 1:**
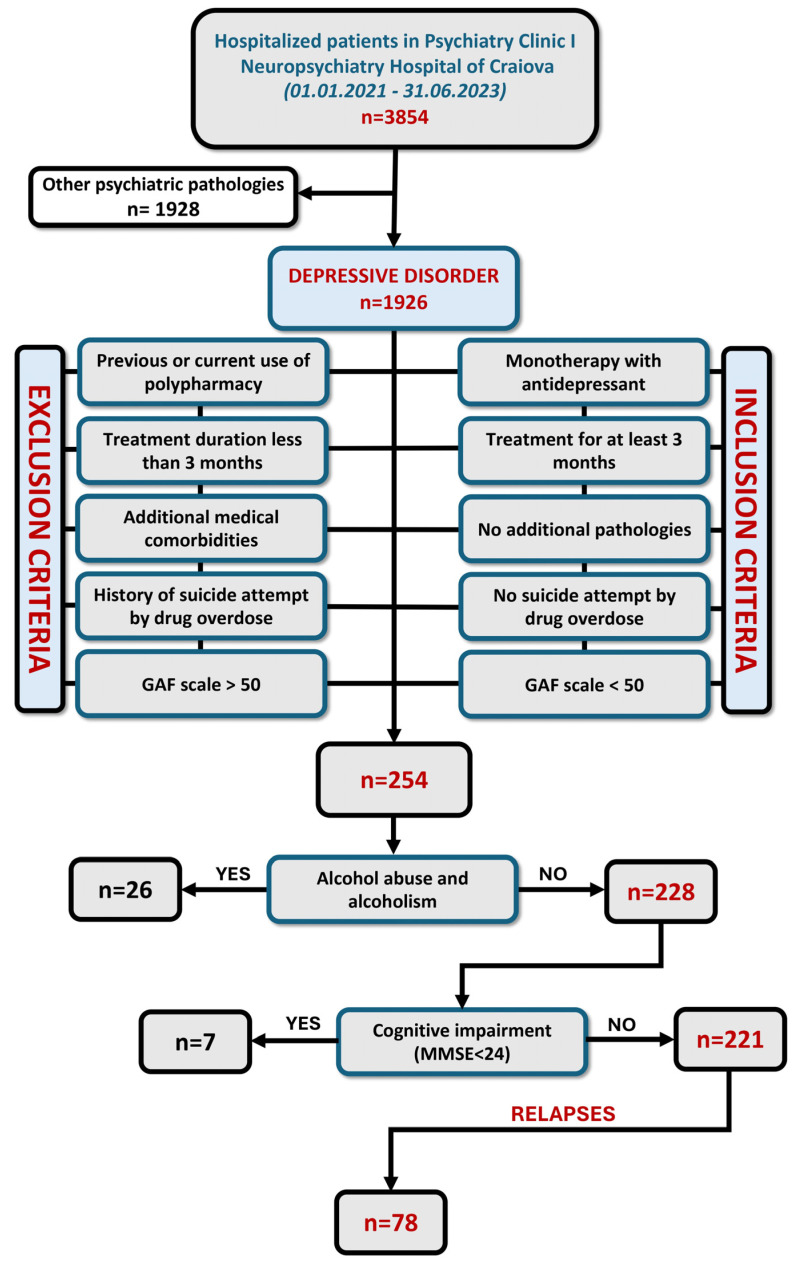
Flow chart of patients with depression that were selected for the study. Out of 1926 patients diagnosed with depressive disorder, 221 were included in the study, after applying the inclusion criteria. Among these, 78 experienced relapse episodes.

Patients with diagnosed major depressive disorder, according to the International Classification of Diseases, Tenth Revision (ICD 10) criteria [49], who received monotherapy with antidepressant treatment for at least 3 months, of either gender, age ≥ 18 years old and without additional pathologies, were included in the study (Figure 1).

Our patients underwent a comprehensive array of medical analyses and investigations in order to exclude other associated pathologies, including hepatitis, diabetes or other liver or endocrinological diseases. By excluding individuals with coexisting medical conditions, we aimed to enhance the accuracy of the study’s findings, attributing them more directly to the effects of moderate alcohol intake rather than the influence of other health conditions. No patient included in the study was hospitalized for a suicide attempt via drug overdose. Twenty-six patients with alcohol abuse and alcoholism and seven patients with significant cognitive impairment were excluded from the beginning. The patients included in our study were patients who described their alcohol consumption as occasional, in moderate amounts, of a maximum of 2 drinks per day (0.22 to 1.00 fl oz alcohol per day), not every day [50]. Cognitive function was assessed through the Mini-Mental State Examination (MMSE) [51,52]. We excluded all patients with a score below 24, indicating possible cognitive impairment. Patients’ functionality was assessed based on the Global Assessment of Functioning (GAF) scale [53] each time they were hospitalized. All patients included in the study had a GAF scale below 50; therefore, the study involved only individuals whose depressive symptoms were severe enough to affect their ability to function effectively.

### 2.3. Antidepressant Treatment

During the study, the patients were treated via monotherapy with one of the following pharmacological classes of antidepressants: selective serotonin reuptake inhibitor(s) (SSRI) (escitalopram, paroxetine, sertraline), selective serotonin-norepinephrine reuptake inhibitor(s) (SNRI) (duloxetine, venlafaxine), atypical tricyclic antidepressants (TCAs) (tianeptine), noradrenergic and specific serotonergic antidepressants (NaSSAs) (mirtazapine) and serotonin antagonist and reuptake inhibitors (SARIs) (trazodone).

### 2.4. Clinical and Biochemical Evaluations

We collected a relevant past medical and psychiatric history, a complete anamnesis, including addictive behaviors and a detailed interview about alcohol consumption, medication received and liver enzyme values: gamma-glutamyl transferase (GGT) (gamma-GT kit, BioSystems, Windsor, CO, USA, 21520), aspartate aminotransferase (AST) (Aspartate Aminotransferase (AST/GOT) kit, BioSystems, 21531), alanine aminotransferase (ALT) (Alanine Aminotransferase (ALT/GPT) kit, BioSystems, 21533) and AST/ALT ratio. The samples were processed with the BioSystems BA400 Smart Efficiency Biochemistry Analyzer.

### 2.5. Statistical Analysis

Data were analyzed with GraphPad Prism 10.1 and Microsoft Excel 2016, and figures were generated with Adobe InDesign 2024. Normality testing was performed using the D’Agostino and Pearson test with an alpha value of 0.05. Differences in means among the groups were analyzed using a Kruskal–Wallis test with multiple comparisons and a two-stage linear set-up, Wilcoxon test, Mann–Whitney test and Kolmogorov–Smirnov test. The difference was considered if the *p*/*q* value was under 0.05. All figures show the mean value and standard deviation (SD). Comparisons use * < 0.05.

## 3. Results

### 3.1. No Differences in Relapse Were Observed in Depressive Patients, Regardless of Liver Enzyme Levels

A total of 221 patients met the inclusion criteria. The study group had an average age of 53.99 ± 8.94 years and a male-to-female ratio of approximately 1:2. Descriptive statistics of the studied population’s liver enzyme levels revealed that the average measured biological parameters were within the acceptable normal range, with the mean GGT value being the only one exceeding the upper limit (40.9 ± 32.1 U/L). Testing the normal distribution of the data, according to D’Agostino & Pearson test, indicated that liver enzyme levels (AST, ALT, AST/ALT ratio, GGT) did not follow a Gaussian distribution (Table 1).

Of the total number of patients, all Caucasians, 72, were treated with SSRIs, 61 with SARIs, 40 with SNRIs, 34 with TCAs and only 14 with NaSSAs (Table 2).

**Figure 2:**
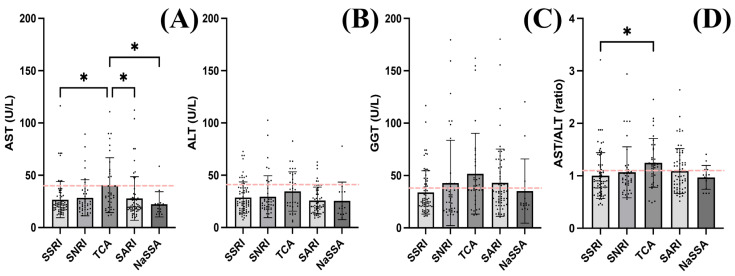
Liver enzyme levels according to treatment received. A Kruskal–Wallis test (two-stage linear step-up procedure of Benjamini, Krieger and Yekutieli) revealed that patients treated with TCAs had higher (**A**) AST levels compared to SSRIs, SARIs and NaSSAs and no difference compared to SNRIs. No differences in (**B**) ALT and (**C**) GGT levels regarding the treatment were observed. (**D**) The AST/ALT ratio was also increased in TCA-treated patients compared to SSRIs. The dashed line indicates the upper normal limit as determined by the protocol for biological parameters adopted locally. The graphs show mean values ± SDs, * q < 0.05.

Within the studied population, no difference in ALT and GGT levels was observed regardless of the treatment (Figure 2B,C). However, when testing AST levels, TCA-treated patients had higher enzyme levels (40.6 ± 26.22 U/L) compared to those treated with SSRIs (26.73 ± 17.23 U/L, *q* = 0.012), SARIs (27.93 ± 20.83 U/L, *q* = 0.012) and NaSSAs (22.27 ± 12.09 U/L, *q* = 0.012) (Figure 2A). No differences were observed between patients treated with TCAs and SNRIs (28.55 ± 17.25 U/L, *q* = 0.1) (Figure 2A). The AST/ALT ratio was also different between patients treated with TCAs (1.24 ± 0.46) compared to SSRI-treated patients (1.01 ± 0.43, *q* = 0.015) (Figure 2D).

Of the 221 patients, 78 had relapsing episodes of depression during the study interval (35.29%). Out of these, 65.38% were female and 34.62% were male. Regardless of the elevated AST, ALT, AST/ALT ratio or GGT levels, we identified no overall risk of relapse, although the higher relapse odds ratio (OR) was in patients with elevated AST levels (1.41, *p* = 0.334). Similar results were observed when investigating the classes of antidepressants used to treat the 78 patients. However, despite not being significant, the highest OR for a relapse was seen for the SNRI-treated patients (1.45, *p* = 0.292) and the lowest for patients with SARIs (0.52, *p* = 0.082).

### 3.2. SNRI Treatment Should Be Avoided in Patients with Any Type of Alcohol Consumption

Although alcoholic patients were excluded from this study, among the 221 patients, some had higher than normal values of GGT levels and AST/ALT ratios, indicating moderate alcohol consumption (Figure 2).

**Figure 3:**
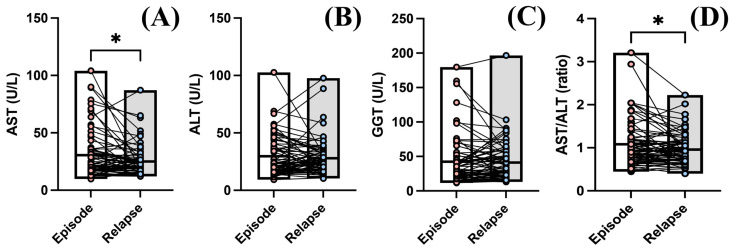
Liver enzyme levels between relapsing episodes of depression. According to a Wilcoxon test, we observed a decrease in the blood levels of (**A**) AST from 30.62 ± 20.98 U/L to 25.02 ± 13.10 U/L (*p* = 0.0217). This phenomenon was not observed for (**B**) ALT and (**C**) GGT levels. (**D**) Between episodes, a decrease in the AST/ALT ratio was observed from 1.083 ± 0.522 to 0.9599 ± 0.3473 (*p* = 0.0216). The graphs show mean values ± SDs, * *p* < 0.05.

While no identifiable risk of relapsing was observed based on blood liver enzyme values, we wanted to see how liver parameters changed between episodes. Surprisingly, in relapsed patients, we observed a decrease in AST levels from 30.62 ± 20.98 U/L to 25.02 ± 13.10 U/L (*p* = 0.021) (Figure 3A). The same was observed in the assessment of the AST/ALT ratio, which decreased from 1.083 ± 0.522 to 0.959 ± 0.347 in the subsequent laboratory analysis (*p* = 0.021) (Figure 3D). ALT and GGT levels did not vary between the two episodes (Figure 3B,C).

Due to these findings and the higher OR in relapses observed for AST levels, we performed a relapse risk analysis based on liver enzyme levels and received treatment (Table 3). When calculating the OR of relapse based on blood values and medication, we estimated that patients with an elevated AST treated with SNRIs had a 9.16 times higher risk of relapse (*p* = 0.028) (Table 3). The same patients with just an elevated GGT had a 5.34 higher risk of relapse (*p* = 0.018). Although some differences were found in the AST/ALT ratio, the OR did not reveal any difference in patient treatment (Table 3). Relapsing patients and improved AST blood levels and AST/ALT ratios compared to the initial episode.

### 3.3. Relapsing Male Patients Exhibid Higher GGT Levels Compared to Those without Any Relapse Episode

**Figure 4:**
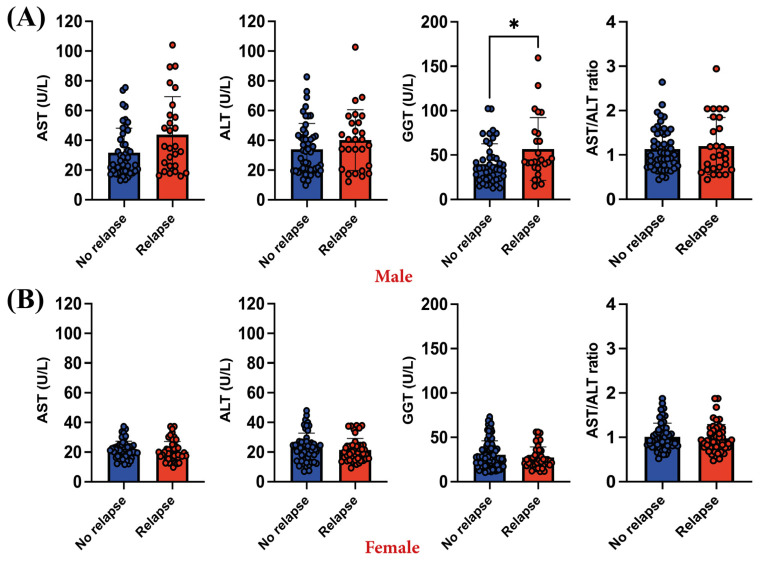
Differences in liver enzyme levels among (**A**) male and (**B**) female patients experiencing relapses compared to those who did not. The graphs show mean values ± SDs, * *p* < 0.05.

After identifying outliers using the ROUT (*Q* = 1%) method, when comparing liver enzyme levels between patients with relapses and those without, the only difference observed in male patients was in GGT levels, in both the Mann–Whitney test (*p* = 0.0203) and the Kolmogorov–Smirnov test (*p* = 0.0244). Relapsing male patients exhibited elevated GGT levels (56.87 ± 35.40 U/L), compared to those without relapse (39.88 ± 22/80 U/L) (Figure 4A). Regarding female patients, no differences were observed (*p* > 0.05) (Figure 4B).

**Figure 5:**
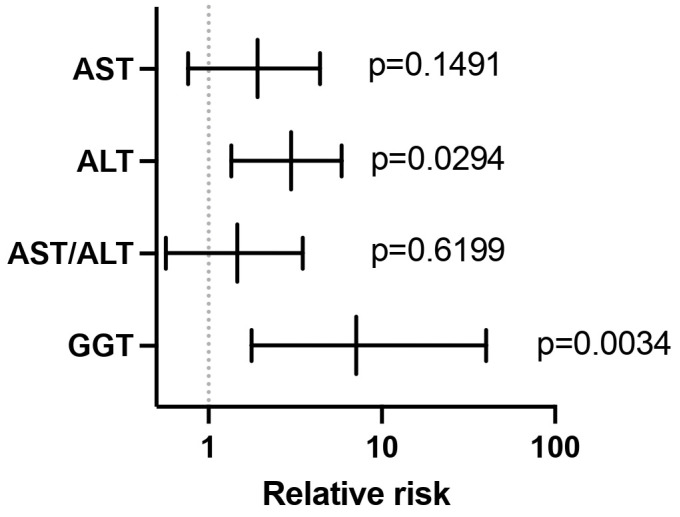
Relative risk of men with higher levels of liver enzymes treated with SNRIs having a relapse compared to women with the same biological status and treatment option, according to Fisher’s exact test. The graph shows the relative risk and the 95% CI.

Except for GGT levels in men (54.93 ± 41.49 U/L), none of the average parameters exceeded the upper normal values. We also wanted to see how elevated liver enzymes in men suffering a relapse are influenced by treatment compared to women, and we checked if enzyme levels in patients receiving certain treatment were different for men and women. After applying Fisher’s exact test, in SNRI-treated patients, no relative risk was found for elevated AST levels or AST/ALT ratios (Figure 5). However, we were able to find that men with higher levels of GGT or ALT, treated with SNRIs, had a higher relative risk of relapse (7.11 for GGT (*p* = 0.0034) and 3 for ALT (*p* = 0.0294)) compared to women with the same treatment (Figure 5).

## 4. Discussion

In order to better understand the effect of moderate alcohol consumption on the outcome of depression, we assessed liver enzymes in patients undergoing treatments with various classes of ADs. The overall relapse rate for the investigated population sample was 35.29%, lower than that reported in previous studies [54,55], with female patients relapsing twice as often as males.

We observed no difference in relapses in depressive patients, regardless of liver enzyme levels. Although patients with alcoholism were excluded from the study, patients with moderate alcohol consumption were not. Among clinicians, it is known that self-reported drinking cannot be taken into account in its entirety, with liver tests being necessary to determine if patients minimize their consumption, and that self-reporting alcohol consumption is highly variable with a majority of them advising for additional objective testing or improving the methodology [56,57,58,59,60]. The combined use of questionnaires alongside GGT level assessments is recommended to ensure a more accurate evaluation of alcohol consumption in patients with depression [61].

It stands to reason that in some patients, there was a strong clinical suspicion of alcohol use, as some of the patients were started on TCA treatment, which is known for its effectiveness in individuals with alcohol intake [62,63,64]. Paraclinical results supported this suspicion, as patients treated with TCA had higher average AST levels, compared to patients treated with other classes of ADs. The clinical suspicion of alcohol consumption was also sustained by increased values of the AST/ALT ratio in patients treated with TCA, compared to those with SSRIs; the ratio was significantly higher. An increase in the AST/ALT ratio is considered highly suggestive that alcohol is the cause of the patient’s liver injury [65,66]. While it will be easy to assume that the alterations in liver enzymes are caused by hepatic toxicity induced by ADs, this is not the case, as under treatment, patients who experienced a relapse displayed lower AST levels and AST/ALT ratios at the time of relapse. This aligns with previous research indicating that individuals undergoing antidepressant therapy usually experience a decrease in alcohol intake [67], with a normalization of hepatic enzyme levels correlated with the reduction in alcohol consumption and effective depression management [68].

Although we were not able to identify any overall relapse differences, we observed that patients with elevated AST who were treated with SNRIs were 9 times more likely to experience depression relapse. The risk was 5 times higher if the same patients had elevated GGT levels. This aligns with previous studies correlating elevated GGT enzyme values with greater negative mood [69], or even different psychiatric comorbidities, including depression [70]. Surprisingly, regarding the AD used, in another study, between 9 and 18% of patients treated with SNRI therapy had relapsing episodes [54]. Although excessive alcohol intake has been reported as an aggravating factor for liver alterations during SNRI treatment [71,72,73], in the present study, moderate alcohol intake did not induce any hepatic cytolysis.

We were able to confirm previous reports regarding the low hepatic impact that SSRI treatment has on depressive patients with moderate alcohol consumption [46,47,74,75]. However, we did not confirm the high relapse rate reported by other studies [54]. Our results align with earlier research reporting that SARI treatment has the lowest relapse risk [54] and establish its efficacy also in terms of moderate alcohol consumption, as previous studies were able to show not only a reduction in depressive and anxious symptoms in patients with a history of alcohol abuse [76]. The observed results could be attributed to well-known effects of SARIs, such as a reduction in insomnia [77], especially since sleep problems are closely related to both MDD [78,79] and alcohol use disorder (AUD) [80]. A limitation of the present study is that we did not properly address insomnia, as many individuals use alcohol in an effort to alleviate it [81] and may experience sleep disturbances upon discontinuing drinking [82].

With a gender ratio of approximately one male to two females suffering from depression, our data align with previous reports sustaining that women are twice as likely to experience MDD compared to men [83,84]. This higher prevalence may be influenced by various factors, including biological, psychological and socio-cultural factors [85,86]. Hormonal fluctuations, such as those related to menstruation, pregnancy and menopause, may contribute to women’s increased vulnerability to depression [87]. Additionally, societal expectations and gender roles may shape how men and women express and cope with emotional distress, potentially leading to the underreporting or misinterpretation of symptoms [88].

Although previous studies have shown that women are also more vulnerable to hepatotoxicity compared to men [74], in our study, GGT levels in men exceeded the upper normal values. While ALT proved to have the most significant association with NAFLD [23], GGT is recognized as a biomarker linked to alcohol consumption [25,26]. With the careful exclusion of alcoholism from the present study, our findings might imply that men underestimated their alcohol intake at the start of the therapy. This aligns with previous research indicating that men tend to understate their alcohol intake [89]. Among men, alcohol consumption is more prevalent as a coping mechanism for stress [90,91,92] and to alleviate depressive symptoms [93]. Although men generally have higher levels of liver enzymes [23,94], studies suggest that women may progress more rapidly from early stages of liver disease to advanced stages [95,96].

Our finding regarding the higher risk of relapse in patients with altered liver enzymes who were treated with SNRIs was observed in male patients. The observed increased risk could be a consequence of the different ratio between men and women enrolled in the study, or it could be caused by a better response of women to SNRI treatment. The latter is not unreasonable, as previous research reported that women can exhibit a more favorable response to serotoninergic antidepressants compared to men [97,98]. However, this gender difference is not universally accepted, as some studies have found no depression-related difference between genders in SNRI treatment [99]. While there are opposing views regarding gender differences in ADs related to gender, future research is needed to establish if these differences are maintained in patients with moderate alcohol intake.

One particular variable to investigate, given the increased vulnerability of older individuals to hepatotoxicity [74,100], should be age. However, improvements in depressive symptoms seem to correlate more with the choice of AD than with the patient’s age [99]. While future studies on moderate alcohol use and depression might also consider the age of the patients, alcohol is the real impediment to treating depression [101,102].

This study has several limitations. Our findings are specifically applicable to the antidepressants utilized in this particular study, and additional research is needed to explore the effects of other antidepressants on both patient outcome and liver function. This is particularly needed as several reports have shown a reduction in the frequency and intensity of alcohol cravings in alcoholic individuals [41,103], with different efficacies in patients with both comorbid MDD and an AUD [40]. Although we could partially validate some of the previous findings, as discussed above, future studies are needed, as the current findings might be influenced by the limited number of patients examined. Furthermore, this analysis focused on patients receiving antidepressants in monotherapy, and it would be beneficial to investigate the outcomes of combined therapy, as well as drug–drug interactions, including the concurrent use of two antidepressants [18], representing a significant concern for liver toxicity.

## 5. Conclusions

Patients with depression, particularly men, often tend to minimize their alcohol consumption, and what they perceive as insignificant may pose an impediment to treating depression. This becomes especially notable when the chosen AD belongs to the SNRI class, potentially increasing the risk of depression relapse compared to other antidepressants.

## Figures and Tables

**Table 1:** Descriptive statistics of liver enzyme levels in the studied population and results of their normal distribution testing according to D’Agostino & Pearson test.

		AST Blood Levels	ALT Blood Levels	AST/ALT Ratio	GGT Blood Levels
Normal Values		1–40 U/L	1–41 U/L	<1.1	1–38 U/L
Descriptive statistics of the studied population	Mean	29.24	28.75	1.07	40.93
	Std. Deviation	20.1	16.4	0.4	32.1
	Minimum	9.8	5.5	0.4	10.4
	Maximum	116.5	102.7	3.2	180.2
	Range	106.7	97.2	2.8	169.8
	Skewness	2.2	1.4	1.6	2.2
	Kurtosis	4.9	2.5	3.7	5.5
Test for normal distribution D’Agostino & Pearson test	K2	110.9	64.6	79.4	114.5
	*p* value	<0.0001	<0.0001	<0.0001	<0.0001
	Passed normality test (alpha = 0.05)?	No	No	No	No

**Table 2:** Descriptive statistics of patients based on the type of antidepressant, age, gender distribution and relapse episodes.

		Total	SSRI	SNRI	TCA	SARI	NaSSA
Included patients	No. of patients	221	72	40	34	61	14
	Mean age (years)	53.99 ± 8.94	53.19 ± 8.15	54.58 ± 8.93	51.50 ± 9.37	54.97 ± 9.72	58.14 ± 6.87
	Gender Distribution (M:F)	76:145	13:59	18:22	18:16	23:38	4:10
No relapse	No. of patients	143	44	23	21	45	10
	Mean age (years)	53.52 ± 9.68	54.32 ± 7.60	54.17 ± 10.50	49.33 ± 10.56	53.87 ± 10.75	58.10 ± 7.34
	Gender Distribution (M:F)	49:94	10:34	9:14	10:11	18:27	2:8
Relapse episodes	No. of patients	78	28	17	13	16	4
	Mean age (years)	54.83 ± 7.36	51.46 ± 8.79	56.35 ± 6.04	55.00 ± 5.87	58.06 ± 5.05	58.50 ± 6.55
	Gender Distribution (M:F)	27:51	3:25	9:8	8:5	5:11	2:2

**Table 3:** Odds ratio for depression relapses according to treatment and biological status.

	SSRI		SNRI		TCA		NaSSA		SARI	
	OR	*p* Value	OR	*p* Value	OR	*p* Value	OR	*p* Value	OR	*p* Value
**Elevated AST**	0.936	0.932	9.167	0.028	1.016	0.983	INC.	INC.	1.253	0.767
**Elevated ALT**	1.442	0.553	2.778	0.201	0.593	0.481	INC.	INC.	0.433	0.445
**Elevated GGT**	1.015	0.977	5.344	0.018	3.000	0.134	INC.	INC.	0.622	0.440
**Elevated AST/ALT**	0.889	0.831	1.545	0.530	0.467	0.297	0.778	0.852	0.750	0.630

## Data Availability

The data presented in this study are available upon request from the corresponding authors.
